# Case of Hydrophobia

**Published:** 1809-01

**Authors:** G. Pinckard

**Affiliations:** Bloomsbury Square


### CASE of HYDROPHOBIA, by Dr. PINCKARD.

WILLIAM WATERS, of Chipping Barnet, Herts, a sawyer, aged 25 years, a strong, healthy man, married, and father of one child, was bitten on the 14th day of September last, close above the upper joint of the little finger of the left hand, by a strange dog, which he met running upon the public road between Barnet and Whetstone. The wound was deep, and the laceration extensive. He applied to Mr. Lloyd, a surgeon at Barnet; and, no suspicion of madness being entertained, the common treatment, as in other recent wounds, was employed. The cure proceeded without any circumstance worthy of particular remark, leaving an eschar about an inch in length. No provocation was given to the dog; nor has any opportunity occurred of ascertaining whether or not he was affected with rabies.

On Friday the 26th of November, seventy-three days from the time of the accident, the man felt slightly indisposed, and returned home from work, without having eaten his usual dinner; but it was observed that he drank a draught of porter. About eight o'clock in the evening he called upon Mr. Lloyd, complaining of a severe pain in his left shoulder, saying he could not raise his arm to his head. Both the surgeon and himself considered it to be rheumatism. A bolus of pulvis ipecacuanhæ compositus was accordingly prescribed, to be taken at bed-time, and he was directed to rub the part with a spirituous embrocation; but, feeling himself much worse after he went to bed, he sent, about eleven o'clock, for Mr. Lloyd to visit him, when he still complained of the pain in the shoulder, adding, that he was distressed likewise with "the wind." Another of the boluses was administered, which he swallowed with difficulty, and he was advised to take some warm wine and water, but he put it away, saying that he could not drink it. Mr. Lloyd felt less satisfied respecting the nature of the disease than when he saw him in the evening, but no suspicion yet arose that it might be hydrophobia.

During the night he remained extremely restless, and groaned so as to disturb the family in the adjoining house; but the pain of the shoulder subsided, leaving, as he expressed it, "a tightness and choaking about the throat," which increased to an alarming degree. Between seven and eight o'clock the following morning Mr. Lloyd repeated his visit, when he found him in a state of extreme agitation, with a sense of constriction about the throat, and great uneasiness and oppression at the epigastric region. His respiration was irregular and convulsive, and he had frequent eructations of flatus. In order to obtain relief from the difficulty of breathing and sense of suffocation, he had placed himself upon his knees and elbows in bed. Some water being offered him to drink, he suddenly started with terror and alarm, was thrown into violent convulsive distortions, looked offended, and said he could not take it.

The nature of the disease being no longer doubtful, Mr. Lloyd had immediate recourse to mercurial friction. About three ounces of the unguentum hydrargyri fortius, mixed with camphire, were rubbed in by three persons upon the extensive surface of the neck and thorax, the patient himself assisting. This process was continued until he felt greatly exhausted. He then begged to be left quiet, saying that he was better. His pulse was at this time languid and feeble. After he was a little rested, about two ounces more of the ointment, mixed with opium, were rubbed into the legs and thighs; the friction being continued until it was interrupted by excessive agitation, and general convulsions.

The violent symptoms of this most dreadful of all human calamities now increased rapidly. Any liquid was an object of perfect horror to him; the moving of it in a basin, pouring it from one vessel into another, splashing it about the room, placing it before his eyes, or even speaking of it, produced inconceivable agitation, accompanied with a peculiar expression of terror, and a dreadful distortion of the whole frame. Some water being presented to him, he was instantly seized with convulsions, sprang up suddenly, and leaped out of bed, throwing himself from the very sight of the basin. At this period of the disease the convulsions recurred in rapid succession; a considerable quantity of frothy saliva issued from his mouth, he uttered hideous and indescribable groans, looked trembling and terrified, and a marked expression of horror* settled upon his countenance. Soon afterwards it was observed that his urine passed involuntarily; he complained

* So strikingly was the expression of horror depicted upon his features, that a gentleman, who was in the room, noticed it as being highly interesting and picturesque; remarking, that if it could have been viewed abstractedly from the sad distress of the scene, it would have been precisely what a spirited painter, desirous of excelling in his art, might have wished to delineate.

plained more and more of the "wind and choaking"; the general agitation and restlessness increased; the convulsions grew stronger and stronger, and the groans and screams louder, and more frightfully distressing.

Between ten and eleven o'clock he was quite outrageous; and the convulsions being so powerful that four people were unable to hold him in bed, it was deemed expedient to have recourse to a straight waistcoat. During the violence of the convulsions, one of the persons, who was holding him, said that he attempted to bite him; but he immediately apologised, observing, that he did not mean to hurt him; and Mr. Lloyd, who witnessed this circumstance, believed it to be accidental rather than intentional.

The cicatrix produced by the wound upon the hand, was examined, and the nature of the malady was openly talked of by the crowd of persons who came into the room; but, instead of feeling any apprehension upon the subject, he would not admit that the disease was in any way connected with the bite he had received. He persisted in calling it "the wind," but expressed himself conscious that he could "never recover". No change could be perceived in the part which had been bitten, except that the scar appeared slightly livid as if it were from cold. It was neither swelled nor inflamed; nor was there any tumor, inflammation, or soreness in the glands of the axilla: but, on being questioned particularly respecting the state of the limb, he remarked that he had felt a sense of cold or numbness in the hand and arm, for two or three days previous to his being unwell; and that he had covered the bitten part again with a "thumb-stall," which he had used for some time after the wound had healed.

The restlessness, terror, extreme agitation, and strong convulsions continued until noon; the convulsions recurring with excessive violence at intervals of only two or three minutes, and from the slightest irritation; mostly from the sight, the sound, or only hearing the name of water. About one o'clock he became more calm, and it was perceived that the horror and aversion to liquids were in some degree diminished. Soon after, he was prevailed upon to swallow two drachms of the tincture of opium.

It was between seven and eight o'clock in the evening of the 27th of November when I first saw him. Messrs Lloyd, Rumbold, Booth, and Morrison, medical practitioners at Barnet, were present. He was then lying in a straight waistcoat, extended upon his back, with his hands and feet fastened to the bedstead. He was tranquil and composed; his countenance was natural, and his intellects undisturbed. To the questions which were asked him, he eplied in a collected and rational manner; and he was sensible of all that passed in the room. His skin was of natural warmth, and covered with a moderate perspiration. The pulse did not exceed 90 in a minute: it was obtuse and undulating. On pressing his wrist with the fingers the artery was perceived to be slightly tremulous. The tongue was moist, and, although whitish, nearly of a natural appearance; the eye looked rather flat and clouded. The convulsions had ceased; the dread of liquids was removed, and he frequently called for water to drink: but he had still a great source of terror and agitation from a peculiar sensibility to currents of air falling upon his skin; and to the impression of odours upon the olfactory organs. The senses of feeling and smelling seemed to be preternaturally increased. He had no pain, but was extremely distressed with flatulency. His respiration very much resembled that of a female in a paroxysm of hysteria. It was accompanied with frequent irregular sighing, and almost constant eructations of wind. On my asking him to describe his feelings, he said, " I am better, much better; I have no complaint but the wind and choaking;" and upon my loosening one of his hands, in order that he might accurately describe the parts most affected by constriction, he pointed distinctly to the throat and epigastrium.

It was distressing to observe the anxiety and the frequency with which he now called for water; yet I observed that he never took it by deliberate drinking, so as to bring the organs of deglutition into any number of successive actions. Each time it was given to him he seized the cup eagerly, both with his lips and his hand, made one convulsive swallow, then hastily pushed away the vessel, saying, if the person who held it chanced to press it longer to his lips, that he gave him " too much," and would " choak" him. Several loud eructations of air usually followed the swallowing of the water, and he remarked, that he drank it because it " broke the wind, and eased" him. For a short time after obtaining this relief his breathing was less disturbed, and he conversed with all the calmness of a person in sound health; but soon the spasmodic feeling about the throat and stomach increased, the respiration was oppressed, and he again called anxiously for " drink, to move the wind," as he expressed it. On my giving him some wine in the water, he said it relieved him more than the the water alone; but he begged that it might not be made strong; observing that if it were he could not swallow it.

Next to his anxiety for frequent drinking, the greatest distress that he suffered proceeded from the opening and shutting of the chamber door; which, indeed, was the most characteristic symptom at this stage of the disease. He was more watchful, regarding the door, than concerning any other object. Whenever it was moved, he started in great agitation, looked terrified, and impatiently called out "the door, the door;" and although he neither saw nor heard it opened, so acutely sensible was he of the slightest current of air, that he instantly knew from his own sensations, when any person entered or left the room. The slightest current of the breath falling upon his face from any person who was speaking to him; air blown from the lips upon his breast, and the fanning of a hat across his chest or throat, produced great agitation, together with convulsive breathing, and a sense of suffocation: but the same effect was not observed from waving a hat across his feet and legs; nor from suddenly sprinkling a few drops of cold water upon his face or thorax. A candle was held near to his eyes, but he expressed no uneasiness from the light of it. He had a dread of any person standing near his face; also of any substance being put in motion near his mouth; and of any thing strong or volatile being applied to his nose. He seemed likewise to have a terror respecting the moving, or in any way disturbing his person. He expressed himself satisfied to lie fastened in the waistcoat; and when his hand was released, said that it gave him no relief. He swallowed the water, lying upon his back, with the head low; and refused to be raised, when it was proposed to lift him up to drink it; he complained of the wind produced by a handkerchief, which was used to wipe the saliva from his lips; and he was greatly disturbed by the smell of a cloth which happened to be placed upon the bed, after being used by one of the persons who had been employed to rub in the ointment with camphire. Once some wine was offered to him, instead of the wine and water, but, when it approached his nose, he suddenly refused it, saying, impetuously, "it is too strong, I cannot drink it." Between nine and ten o'clock he requested to see his wife and child, when he tenderly pressed the hand of the mother, but anxiously desired her not to put the child near his mouth; manifestly, not from any apprehension of injuring the child, but from a dread of the air being disturbed about his face.

The

The tincture of opium was directed to be repeated every hour, in doses of half a drachm, combined with a scruple of the oleum succini rectificatum. He took it three times, but it did not appear to have any influence whatever upon the symptoms, and he complained that it was strong, and made him worse.

At midnight, upon observing a person in the room eating roasted apples, he requested to have some, and ate nearly two of them, with seeming gratification. He then said that his stomach was "restored," and, feeling as if he could eat something more, desired to have a "beef-steak for supper." This was accordingly prepared, and he chewed two or three morsels, but did not swallow them.

About one o'clock in the morning of November 28th, the high susceptibility, and the dread of currents of air left him, and he desired to have the door and the window set open. He now remarked that he was much worse; requested to be released from the confinement of the waistcoat; and said, impressively, that he should "soon be gone." His eagerness for water became quite insatiable, and although his stomach now began to reject it by vomiting, he called for it incessantly. On one of the by-standers asking him if he were not afraid that so much water might do him harm, he replied "No, I feel it running off as I drink it;" proving, that although his urine passed involuntarily, it was not without consciousness. He likewise desired to have cold water applied to his nose; and his impatience for it increased to such a degree that two persons found full employment in wetting his nostrils, and giving him water into his mouth. Before two o'clock he expressed a similar eagerness and impatience for air, asked those near the bed to blow upon him, and desired every person to stand away from the door, that he might feel the cold current. He remained perfectly sensible (as he had been throughout the whole of the disease), and without any return of convulsions, until nearly *three o'clock*, when he expired; his last moments being marked with calmness and composure.

Very soon after death a number of dark red, or livid blotches appeared about the throat and clavicles; and the abdomen became tense, and much enlarged.

### Appearances on Dissection.

On opening the head, the dura mater adhered so strongly to the cranium, that great force was required to separate them. The whole surface of this membrane appeared ed in a state of unusual dryness, and was more free than is common from small red points, or exudations of blood. The vessels of the pia mater were not over-charged with blood.

The brain was remarkably close and firm in its texture. A peculiar dryness was observed throughout the whole of its substance. The cerebrum appeared beautifully white, and had not those numerous red points which are usually observed. When cutting the cortical and medullary portions they both opposed a strong resistance to the knife, they also preserved their form under considerable pressure from the finger. A small quantity of colourless fluid was contained in the ventricles.

On cutting through the integuments and muscles of the thorax, to turn them back, for the purpose of exposing the ribs and sternum, the whole fleshy substance was observed to be in a state of unusual dryness.

The viscera of the thorax had a healthy appearance. The lungs were fully distended with air. There was a general dryness upon the surface of the pleura. The pericardium contained about half an ounce of fluid.

The posterior part of the tongue, the outer surface of the epiglottis, and the whole of the pharynx, exhibited strong marks of inflammation: some degree of redness was also observable, although not so conspicuous within the larynx, and upon the surface of the trachea and œsophagus. At the lower part of the œsophagus, about half an inch from the cardiac orifice of the stomach, was an eroded spot, nearly the size of a shilling, assuming an appearance as if the inner coat had been separated and shrivelled up by scorching.

The stomach and intestines were much distended with flatus. Their exterior coats, also the peritonæum covering the other parts of the cavity of the abdomen, and likewise the diaphragm, were in a state of dryness similar to the pleura. The rugæ of the inner coat of the stomach were numerous, large, and very distinct. A few inches below the cardia was a fulness of the vessels of the villous coat, which caused a spotted and circumscribed redness about three or four inches in diameter.

The liver and spleen were of a light or ash-coloured hue; in other respects of a healthy appearance.

The general dryness which prevailed in the fibres of the muscles, within the substance of the brain, and upon the membranous surfaces, extended likewise to the omentum, which which, when pressed in the hand, felt like a loose net of packthread.

It is proper to remark, that the stomach, the œsophagus, and the trachea, were not only carefully inspected by Mr. Lloyd, Mr. Booth, and myself upon the spot, but that they were taken from the body and brought to London, where they were further examined by Mr. Blair and Mr. Dixon, who are much in the habit of inspecting bodies by dissection, and that both these gentlemen, without any communication with each other upon the subject, favored me with a written statement of the appearances they observed, previous to their receiving any intimation that the parts were taken from a person who had died of hydrophobia.

These parts were also examined several successive days, after being immersed in water. The redness of the pharynx was darker and stronger, and assumed a livid hue, as the membrane became corrugated ; but the redness of the membranes lining the trachea and œsophagus, went off soon after the parts were put into water. There was not the slightest appearance of coagulum, exudation, or adventitious membrane, in any part of the pharynx or larynx ; nor throughout the whole extent of the œsophagus or trachea.

The body was examined twenty-nine hours after death.

The disease continued about thirty-eight hours from the time when the man first became sensible of indisposition.

Observing the progress of the symptoms, as they occurred in this case, the disease might be divided, with tolerable accuracy, into several distinct periods, or stages, viz.

1. A sensation of cold and numbness about the wound, and throughout the hand and arm — during two or three days.

2. A severe pain of the shoulder, with undefined general disposition — about ten hours.

3. Horror of liquids, with violent convulsions and distortions — fourteen or fifteen hours.

4. Comparative tranquillity, with a desire for water, and a dread of currents of air — nearly twelve hours.

5. An insatiable craving for air and water — between two and three hours.

G. PINCKARD.

